# Vasculoprotective Effects of Vildagliptin. Focus on Atherogenesis

**DOI:** 10.3390/ijms21072275

**Published:** 2020-03-25

**Authors:** Michał Wiciński, Karol Górski, Eryk Wódkiewicz, Maciej Walczak, Magdalena Nowaczewska, Bartosz Malinowski

**Affiliations:** 1 Department of Pharmacology and Therapeutics, Faculty of Medicine, Collegium Medicum in Bydgoszcz, Nicolaus Copernicus University, M. Curie 9, 85-090 Bydgoszcz, Poland; 2 Department of Otolaryngology, Head and Neck Surgery, and Laryngological Oncology, Faculty of Medicine, Ludwik Rydygier Collegium Medicum in Bydgoszcz, Nicolaus Copernicus University, M. Curie 9, 85-090 Bydgoszcz, Poland

**Keywords:** vildagliptin, endothelium, inflammation, atherogenesis, pathways, pharmacology

## Abstract

Vildagliptin is a representative of Dipeptidyl Peptidase-4 (DPP-4) inhibitors, antihyperglycemic drugs, approved for use as monotherapy and combination therapy in type 2 diabetes mellitus. By inhibiting enzymatic decomposition, DPP-4 inhibitors increase the half-life of incretins such as GLP-1 (Glucagon-like peptide-1) and GIP (Gastric inhibitors polypeptide) and prolong their action. Some studies present results suggesting the anti-sclerotic and vasculoprotective effects of vildagliptin reaching beyond glycemic control. Vildagliptin is able to limit inflammation by suppression of the NF-κB (nuclear factor kappa-light-chain-enhancer of activated B cells) signaling pathway and proinflammatory agents such as TNF-α (tumor necrosis factor α), IL-1β (Interleukin-1β), and IL-8 (Interleukin 8). Moreover, vildagliptin regulates lipid metabolism; attenuates postprandial hypertriglyceridemia; and lowers serum triglycerides, apolipoprotein B, and blood total cholesterol levels. This DPP-4 inhibitor also reduces macrophage foam cell formation, which plays a key role in atheromatous plaque formation and stability. Vildagliptin reduces vascular stiffness via elevation of nitric oxide synthesis, improves vascular relaxation, and results in reduction in both systolic and diastolic blood pressure. Treatment with vildagliptin lowers the level of PAI-1 presenting possible antithrombotic effect. By affecting the endothelium, inflammation, and lipid metabolism, vildagliptin may affect the development of atherosclerosis at its various stages. The article presents a summary of the studies assessing vasculoprotective effects of vildagliptin with special emphasis on atherogenesis.

## 1. Introduction

The prevalence of diabetes mellitus type 2 (DM2) is constantly increasing worldwide at an alarming rate. According to the World Health Organization, nowadays, about 422 million people globally are suffering from this condition [1]. Diabetes confers about a two-fold excess risk for a wide range of vascular diseases such as arterial stiffness, hypertension, and atherogenesis which are the causes of stroke, heart attack, retinopathy, and diabetic foot [2]. In recent years, there have appeared interesting postulates that vildagliptin may display a vasculoprotective properties beyond glycemic control (Table 1). Vildagliptin is a representative of Dipeptidyl Peptidase-4 (DPP-4) inhibitors which are registered and effectively used in DM2 therapy. These class of drugs was designed to increase the half-life of incretins such as GLP-1 (Glucagon-like peptide-1) and GIP (Gastric inhibitors polypeptide) and to prolong their action [3]. Under hyperglycemic state, they stimulate glucose-dependent insulin secretion from pancreatic β-cells and also inhibit the secretion of glucagon from α-cells [4]. Furthermore, they slow down the rate of absorption of nutrients into the bloodstream by delaying gastric emptying. It prolongs the satiety time and, in consequence, restriction of food intake [5]. Interestingly, GLP-1 is also responsible for the activation of some pro-satiety factors such as peptide YY [6]. The abovementioned antihyperglycemic action of vildagliptin lead to a reduction in fasting blood glucose, postprandial glycemia, and HbA1c plasma levels [7]. 

Vildagliptin (1-((3-hydroxy-1-ada-mantyl) amino) acetyl)2-cyano-(S)-pyrrolidine) at a dose 50 mg is rapidly and well absorbed with an absolute oral bioavailability of 85% [8]. Peak plasma concentration after oral administration in the fasting state is observed at an average of 1.5h [9]. High-fat meals delay the absorption rate by 45 min [10]. Excretion is rapid with over 90% excreted within 48 h. After a single oral administration, 100 mg dose of vildagliptin as 85% of the dose was excreted in the urine and 15% in the feces [11]. Cytochrome P450 enzymes do not metabolize vildagliptin to any quantifiable extent, and vildagliptin inhibitor does not induce these enzymes. No dose adjustment is necessary in elderly patients. Vildagliptin should not be used in patients with hepatic impairment. Moderate or severe renal dysfunction requires dose adjustment [12]. 

## 2. Vascular Endothelium

Vascular endothelium is the continuous cellular lining of inner layer of the vascular wall. It constitutes a physical barrier between blood and tissues and plays a crucial role in providing the proper hemostatic balance [35]. Under physiological conditions, it prevents thrombosis via anticoagulant and antiplatelet mechanisms. Endothelial cells synthesize nitric oxide and other factors involved in maintaining proper vascular tone, blood pressure, and local blood flow [36]. The layer is a rich source of ROS (reactive oxygen species), which play a dual role in the cardiovascular system. In moderate concentrations, they have an important signaling function; however, their overproduction is detrimental to the body [37]. Excessive increase of mitochondrial ROS is involved in the development of electrical instability events responsible for sudden cardiac death and mediates heart remodeling in chronic heart failure [38]. Normally functioning endothelium participates in the body’s inflammatory and immunological reactions and regulates cell growth [39]. Various conditions such as hyperglycemia, hypertension, hyperlipidemia, inflammation, hypercholesterolemia, hyperhomocysteinemia, or tobacco addiction disturb endothelial homeostasis and contribute to the development of endothelial dysfunction [40]. Progression of these risk factors underlie the development of atherosclerosis and cardiovascular diseases [41]. Liu et al. (2019) demonstrated that vildagliptin promotes NO (nitric oxide) production and restores physiological ROS synthesis deregulated under diabetic conditions. Vildagliptin also improves diabetes-induced mitochondrial dysfunction. Drp1 and Fis1 (dynamin-related proteins) are essential regulators of mitochondrial fission. Diabetic state is characterized by significantly increased expression of Drp1 and Fis1 [17]. The usage of Drp1 inhibitor mdivi¬1 (mitochondrial division inhibitor 1) () inhibits cardiomyocyte cell death after cardiac arrest. Mdivi-1 preserves the postischemic LV function by preventing mitochondria fragmentation and increased cell viability [42]. Animal studies [43,44,45] showed that DPP-4 inhibitors like vildagliptin reduce infarct size and have a protective effect on heart function after HF. However, human studies have not yet confirmed these observations [46]. Vildagliptin has the ability to reduce the expression of Drp1 and Fis1 and, as a result, mitigates mitochondrial fragmentation induced by hyperglycemia. Decreased expression and activation of Drp1 has been probably caused by increased phosphorylation of AMPK (5’AMP-activated protein kinase) and its target acetyl-CoA carboxylase induced by vildagliptin [17]. In another study performed by Oeseburg et al. (2010), the DPP-4 inhibitor significantly increased GLP-1 levels and limited reactive oxygen species-induced senescence of endothelial cells. GLP-1 induces cAMP (3’,5’-cyclic adenosine monophosphate) production and downstream PKA (Protein Kinase A) phosphorylation. GLP-1 activates the cAMP response element-binding transcription factor (CREB) in cAMP/PKA-dependent manner. Molecular analysis revealed that GLP-1 acting through CREB can induce the oxidative defense genes HO-1 (Heme oxygenase 1) and NQO1 (NAD(P)H dehydrogenase (quinone 1)) [19]. HO-1 provides cytoprotective effects and plays an important role in the development of oxidative and age-related disorders [47]. Using the experimental pancreatectomy-induced vascular dementia model, Jain et al. (2015) presented a wide range of endothelial protective effects caused by vildagliptin. DPP-4 inhibitors significantly attenuated a decrease in serum NO level and improved endothelial dependent relaxation. Moreover, it significantly reduced ROS production by mitigation of the increased levels of calcium, myeloperoxidase (MPO), and aortic superoxide anion as well as a considerable decrease in glutathione (GSH). Administration of vildagliptin by restoring the balance of oxidants/antioxidants improves endothelial function and significantly attenuates impairment of learning and memory [23]. It seems interesting to further evaluate the possible benefits of treatment with selective DPP inhibitors in vascular dementia. Apart from ROS overproduction, further factors correlating with vascular injury are diminishment of circulating endothelial progenitor cells (EPCs) and increased plasma (C-term) stromal cell-derived factor 1α (SDF-1α) [48,49]. SDF-1α is a substrate of DPP-4; hence, DPP-4 inhibitors may increase SDF-1α level by restraining DPP-4-mediated enzymatic decomposition [50]. SDF-1α is a pivotal mediator of stem cell mobilization, which takes part in the homing of endothelial progenitor cells from bone marrow to areas of vascular injury and ischemic sites in order to promote angiogenesis and repair [51]. The effects of SDF-1α appeared to be beneficial in ischemia and restenosis [52,53]. On the other hand, the role of SDF-1α in atherogenesis might be completely different. SDF-1α released by the atherosclerotic plaques stimulates receptors of inflammatory cells such as monocytes and macrophages [54]. As a consequence, the plaque formation process accelerates [55]. In the Framingham Heart Study of 3359 participants (10% of individuals with diabetes), high plasma SDF-1α were directly and independently associated with the risk of heart failure and 10-year all-cause mortality [56]. Two independent randomized double-blind control studies [14,32] in small groups of DM2 patients provide interesting evidence for the effect of vildagliptin on SDF-1α. Although DPP-4 degrades SDF-1α and its blockade should be associated with an increase in SDF-1α, the administration of vildagliptin resulted in a significant decrease in circulating SDF-1α. We can speculate that decreased circulating stem cells in DM2 might induce plasma SDF-1α, and administration of DPP-4 inhibitors decreased it by stem cell mobilization. Dei et al. (2017) noted an increase in the number of EPCs after vildagliptin treatment, what may support this hypothesis [14]. In summary, by lowering SDF-1α levels and by stimulating EPCs, vildagliptin may have an impact on slowing the progression of atherosclerosis by reducing inflammation and by repairing endothelial injuries. However, the ambiguous effect of DPP-4 inhibitors on SDF-1α requires further evaluation.

## 3. Inflammation

Recently, several studies implicate subclinical chronic inflammation as an important pathogenetic factor in the development of insulin resistance and diabetes type 2 [57]. The mechanisms involved in the progression of inflammation include overproduction of proinflammatory cytokines, hyperactivation of vessels via NF-κB (nuclear factor kappa-light-chain-enhancer of activated B cells), increased expression of cyclooxygenase and inducible nitric oxide synthase and unbalanced pro-/anti-inflammatory expression microRNA [58]. Systemic inflammation leads to neutrophil extracellular trap activation and release and may initiate damage to the endothelium. Monocytes and T cells infiltrate the lesion. After that, monocytes differentiate into macrophages. These cells proliferate to sustain their population and secrete pro-inflammatory mediators such as TNF-α (tumor necrosis factor α), IL-6 (Interleukin 6), and IL-18 (Interleukin 6) and thus lead to vascular damage [59,60,61]. Lee et al. (2016) demonstrated an alleviation of inflammatory response after administration of vildagliptin. By downregulation of TRLs (toll-like receptors), it suppressed the increased expression of inducible nitric oxide synthase (iNOS) and phosphorylated JNK (pJNK), activation of the NF-κB pathway, and the resulting production of pro-inflammatory cytokines [13]. NF-κB is considered to be the main intracellular inflammatory pathway mediating the majority of vascular inflammatory responses [62]. High-glucose-induced NF-κB activity inhibits the migration of endothelial cells, which delays the wound healing process and repair lesion in atherosclerotic arteries [63]. Inhibition of the NF-κB pathway attenuates the proliferation and growth of vascular smooth muscle cells, of which the hyperactivity promotes the formation of atherosclerotic plaques [64]. Moreover, suppression of EP2 (prostaglandin E receptor subtype 2) and TNF-α, the NF-κB activators, reduced macrophage infiltration and intracranial aneurysm formation and progression [65]. Elevation of some NF-κB activators, such as osteoprotegerin are associated with elevated mortality, especially due to cardiovascular diseases [66]. Ji et al. (2019) observed a decrease of endoplasmic reticulum (ER) stress markers *GRP78* and *CHOP* after vildagliptin administration to diabetic mice. ER stress/NF-κB pathway suppression inhibits vascular smooth muscle cells proliferation and reduces stenosis of the injured carotid artery [26]. Zhang and colleagues (2018) reveal that the cellular protective effects mediated by vildagliptin involve suppression of NF-κB signaling. They noted decreased production of the intercellular cell adhesion molecule-1 (ICAM-1) and monocyte chemotactic protein 1 (MCP-1), which may stimulate leukocyte recruitment and adhesion to the endothelial wall under pro-inflammatory state. Furthermore, administration of a DPP-4 inhibitor leads to a reduction of vascular inflammatory factors, including TNF-α and IL-8 (interleukin-8) [15]. This molecules act proinflammatory and proatherogenic, increase vascular permeability, stimulate adhesion molecules, and promotes leukocyte arrest [67]. The TNF-α/NF-κB/IL-8 pathway is involved in the regulation of neoangiogenesis [68,69]. Neoangiogenesis of atherogenic arteries increases the local flow of nutrients and oxygen and may thereby promote plaque progression and remodeling. New, immature, leaky, and fragile neocapillaries promote leukocytes infiltration and increase the risk of intraplaque hemorrhages, which can lead to plaque instability and rupture [70]. Plaque rupture unleashes the fatty core, and its high content of tissue factor provide a powerful substrate for the activation of the coagulation cascade. Depending on the size, the formed thrombus may be clinically silent or may cause a critical restriction of the blood flow and acute ischemic event [71]. Coagulation cascade disorders, especially hyperreactive platelets and impaired fibrinolysis, are also a significant problem in patients with DM2 [72]. Interestingly, Khan et al. (2015) reported a significant increase of coagulation biomarkers, including activated partial thromboplastin time (aPTT); prothrombin time (PT); and decrease of proinflammatory factors concentration such as NO, C-reactive protein (CRP), and TNF-α in DM2 rats after 3 weeks of vildagliptin treatment. The most favorable results were obtained when vildagliptin and pioglitazone were used simultaneously. However, vildagliptin alone might prove as a promising therapy for DM-linked thrombosis marked by inflammation and hypercoagulation. Vildagliptin also caused a reduction in cholesterol and triglyceride concentration [22]: Free fatty acids (FFAs) arising mainly from triglycerides and cholesterol. FFAs induce expression of proteins of the NLRP3 inflammasome to enhance production of interleukin-1β (IL-1β) and interleukin-18 (IL-18), which impair endothelial function [73,74]. AMPK (5’ AMP-activated protein kinase) plays a critical role regulating dyslipidemia and improving endothelial function. Activation of AMPK inhibits the production of reactive oxygen species, ER stress, and Nicotinamide adenine dinucleotide phosphate (NADPH) oxidase and increase the bioavailability of nitric oxide. Consequently, it limits pro-inflammatory factors production induced by dyslipidemia and hyperglycemia [75]. Furthermore, AMPK may downregulate FFA-induced increases in NF-κB transactivation [76]. Treatment of endothelial cells with vildagliptin reverses FFA induction: reduced levels of GSH (glutathione), elevated expression of NAPHD oxidase protein, released cellular LDH (lactate dehydrogenase), and generated ROS. Qi et al. (2019) showed that vildagliptin suppresses FFA-induced expression of proteins of the NLRP3 inflammasome complex and mitigates inactivation of the AMPK pathway. It inhibits the production of two major cytokines of the NLRP3 inflammasome: IL-1β and IL-18 [16]. Patients with diabetes and atherosclerosis are especially prone to high levels and persistent increase in IL-1β [77]. Interleukin-1β stimulates the synthesis of numerous secondary inflammatory mediators and induces its own production, which is the key step in the pathogenesis of many auto-inflammatory disease [78]. Ridker et al. (2017) conducted double-blind, randomized study involving 10,061 patients with previous myocardial infarction and a high-sensitivity C-reactive protein. The researchers assessed the effect of canakinumab (IL-1β antibody) on reducing the risk of cardiovascular disease by ameliorating inflammation. Three months of treatment with cenakinumab 150 mg daily resulted in a significantly lower rate of recurrent cardiovascular events than placebo [79]. Interestingly, Younis et al. (2017) observed IL-1β-suppressing effect after treatment with vildagliptin. The addition of vildagliptin to metformin treatment in patients with DM2 and coronary artery disease leads to reduction of hsCRP concentration and suppression of the IL-1β elevation compared to only metformin treatment [33]. The emerging role of inflammation in atherogenesis pathophysiology inclines to increasing interest in targeting inflammation to improve prevention and control of the disease. It seems to be reasonable that future research should focus on a model of combined suppression for various inflammatory response pathways [80].

## 4. Lipids Metabolism Disorders

Lipids metabolism disorders have been shown to be strongly related to the development of atherosclerosis [81]. Population studies have demonstrated that elevated levels of low-density lipoprotein (LDL) cholesterol and apolipoprotein B (Apo B) 100, the main structural protein of LDL, are directly associated with risk for atherosclerotic cardiovascular events [82]. Hyperlipidemia and hyperglycemia lead to increase oxidative damage. Oxidized LDL (Ox-LDL) becomes a strong chemoattractant and enhances the accumulation of massive intracellular cholesterol deposits. Ox-LDL is attracted by monocyte derived macrophages. They are involved in the formation of foam cells constituting the core of core of the atherosclerotic plaque [83]. Clinical studies prove that lipid lowering therapy by statins, ezetimibe, or PCSK9 inhibitors significantly improve CV outcomes [84]. Multicenter, randomized, double-blind control study “REDUCE-IT” carried out on 8179 patients with elevated fasting triglyceride level revealed that addition 2 g of icosapent ethyl (triglicerydes lowering drug) to statin therapy significantly lowered the risk of major ischemic events, including cardiovascular death than with placebo [85]. Noguchi et al. (2015) demonstrated that a single administration of vildagliptin in dose of 50 mg attenuates postprandial endothelial dysfunction and hypertriglyceridemia in healthy, normoglycemic individuals. Postprandial hypertriglyceridemia impairs endothelial function and plays an important role in the development of atherosclerosis [28]. To explain the mechanism of vasculoprotective properties of vildagliptin related to lipid lowering effect and improved endothelial function, Zhang et al. (2018) performed the experimental study on diabetic rats. Twelve weeks of treatment with vildagliptin significantly reduced blood total cholesterol and blood glucose and attenuated endothelial dysfunction. Vildagliptin downregulated the angiopoietin-like 3 (Angptl3) and betaine-homocysteine S-methyltransferase (Bhmt) expression and activated paraoxonase-1 (Pon1) in the aorta of diabetic rats [25]. Angptl3 is a key regulator which can inhibit lipoprotein lipase (LPL) responsible for hydrolyzes triglycerides (TGL) to free fatty acids (FFA) [86]. Angptl3 levels are increased in obesity and T2D patients compared with healthy individuals [87]. Patients with a loss-of-function mutation of Angptl3 are characterized by low plasma total cholesterol (TCL), TGL, HDL, and LDL [88]. Using Angptl3-specific antibodies in mice and monkey lead to reduced plasma TGL [89]. Bhmt by re-methylation can reduce homocysteine levels, of which the high concentration has been recognized as an independent risk factor for the development of cardiovascular disease [90]. It was assumed that Bhmt expression should increase after using vildagliptin; therefore, this phenomenon requires insightful analysis in the future. The last findings presented by the researchers are related to the elevated Pon1 expression. Pon1 is an enzyme possessing antioxidant functions which take part in removal of harmful Ox-LDL [91]. Decreased Ox-LDL concentration lowers the cholesterol accumulation, foam cell formation, and atherosclerotic lesions [92]. Overexpression of Pon1 decreases the oxidative stress and atherosclerotic lesions [93], whereas Pon1 deficiency promotes LDL oxidation and atherosclerotic lesions [94]. These findings may demonstrate the vasoprotective pathway of vildagliptin in vivo. Based on the mentioned study, we can assume that Angptl3 and Pon1 may constitute proposal of novel molecular targets for antiatherogenic drugs. Nevertheless, the lipids-lowering properties of vildagliptin are not unequivocal. Terasaki et al. (2012 and 2013) in both studies conducted on E-null apolipoprotein mice (Apoe (–/–)) did not observe any changes in serum cholesterol levels after vildagliptin treatment [20,21]. Nonetheless, vildagliptin is able to suppress the macrophage foam cell formation. Wang et al. (2016) indicated that reduced macrophage foam cell formation by TLR-4 (toll like receptor 4) inhibitor suppresses the progression of atherosclerosis in apolipoprotein E-deficient mice [95]. Macrophage foam cells play a key role in plaque formation and stability. They promote the recruitment of more monocytes and immune cells and proliferation of smooth muscle cells and fibsroblasts into the subendothelial space [96,97]. Moreover, foam cells produce a number of chemoattractant and proliferation factors [98,99]. Proinflammatory macrophage foam cells exhibit increased secretion of proinflammatory cytokines and susceptibility to apoptosis and significantly reduced secretion of anti-inflammatory cytokines [100]. Impaired atheroprotective functions, including the cholesterol efflux and efferocytosis of apoptotic macrophages, leads to secondary necrosis and enlargement of the necrotic core rich in oxidative and inflammatory components. The progressive thinning of the fibrous cap makes it more prone to rupture [101,102,103]. Exposure of thrombotic components to platelets and procoagulant factors leading to thrombus formation and clinical events. The progression of atherothrombotic disease is characterized by the elevation of numerous coagulation parameters, including the plasminogen-1 activator inhibitor (PAI-1) [104]. PAI-1 is mainly produced by the endothelium but may be also secreted by adipose tissue [105]. Increase in the serum level of PAI-1 has been reported to be related to increase in the serum levels of triglyceride strongly related to progression of arteriosclerosis [106]. PAI-1 deficiency protects against atherosclerosis progression in the mouse carotid artery [107]. Research of Tani et al. (2015) in DM2 patients indicated significant decreased PAI-1 level, serum TGL, and Apo B concentration after vildagliptin treatment [29]. A systemic review of 38 articles evaluating PAI-1 levels in 11,557 patients revealed that elevated plasma PAI-1 antigen levels are associated with higher risk of death, myocardial infarction, or cerebrovascular accident [108]. 

The contradictory results regarding lipid-lowering properties of vildagliptin may result from significant different methodologies. Therefore, it seems to be obvious that the lipid-lowering properties of vildagliptin require multidimensional verification regarding especially both the dosage and the time of treatment.

## 5. Vascular Dilatation and Blood Pressure 

Hypertension is the most important modifiable risk factor for all-cause morbidity and mortality worldwide and is associated with an increased risk of coronary heart disease, arrythmias, heart failure, cerebrovascular disease, peripheral artery disease, and renal failure [109,110]. Hypertension occurs in more than 50% of patients with DM2 and contributes to the development of both micro- and macro-vascular diseases [111]. Elevated blood pressure (BP) causes mechanical injury to endothelial cells. Concomitant endothelial dysfunction caused by other risk factors, e.g., persistent inflammation and hypercholesterolemia, leads to vasoconstriction and to an increase in BP [112]. Double-blind, randomized, controlled trials performed on over 2000 previously drug-naive DM2 patients revealed multidirectional benefits of vildagliptin treatment. After 24 weeks of treatment at doses of 50 mg once or twice daily, Evans et al. (2016) observed significant reduction of systolic and diastolic BP. They also noted the decrease of fasting triglycerides, very low-density lipoprotein cholesterol, and low-density lipoprotein cholesterol whereas high-density lipoprotein cholesterol increased [30]. The recent study showed that lowering BP and lipid levels together significantly reduces the incidence of cardiovascular events and is more favorable compared to the normalization of only one parameter [113]. Duvnjak et al. (2016) revealed that, apart from affecting lipid profile and blood pressure, vildagliptin may improve hsCRP levels and augmentation Index (AI), which is effective marker of arterial stiffness (AS) [31]. The obtained results are consistent with Ott et al. (2014), who also postulated that vildagliptin may improve AS [114]. On the other hand, Zografou et al. (2015) did not observe any effect of vildagliptin on the improvement of AS but noted a decrease in hsCRP and additionally an improvement in β-cell function after treatment [115]. Arterial stiffness impairs relaxation of blood vessels and contributes to an augmentation of the blood pressure. Increased AS has been shown to correlate with cardiovascular morbidity and mortality [116,117]. Decreased vasodilatation in coronary arteries with established atherosclerosis induced vasoconstriction may result in reduced myocardial perfusion and myocardial ischemia [118]. Research suggests that vildagliptin is able to improve vasodilation in a variety of ways. In the van Poppel et al. (2011) study, 4 weeks of treatment with vildagliptin results in greater vasodilatation response to intra-arterially acetylcholine administration compared to acarbose [27]. Acetylcholine stimulates endothelial muscarinic receptors, thereby activating NO synthase. This results in the endothelial release of NO, causing vasodilatation [119]. Interestingly, administration of sodium nitroprusside, which also stimuli NO synthesis, did not cause any significant response of endothelium [120]. Koyama et al. (2016) also observed that acetylcholine induces endothelium-dependent relaxation only in the vildagliptin group but did not modify the endothelial Ca^2+^ level [24]. Additionally, vildagliptin reduces intimal hyperplasia in vein grafts, which may affect enhanced reactivity of vein bypass grafts and may prolong long-term success of vascular interventions [121]. However, the vasodilatory effect is not limited to regulating NO levels. Seo et al. (2019) analyzed the molecular mechanisms of vildagliptin-induced vasodilation. They observed that application of the voltage-dependent K^+^ channel inhibitor or sarcoplasmic/endoplasmic reticulum Ca^2+^-ATPase (SERCA) inhibitor significantly reduced the vasodilatory effects of vildagliptin. Pretreatment with Ca^2+^-activated K^+^ channel blocker; ATP-sensitive K^+^ channel blocker; or adenylyl cyclase, guanylyl cyclase, protein kinase A (PKA), and protein kinase G (PKG) inhibitors does not influence vasodilatory effects of vildagliptin. Based on obtained results, the authors concluded that vildagliptin induced vasodilation via activation of Kv channels and the SERCA pump; however, other K^+^ channels and PKA/PKG-related signaling are not involved in this process [18]. Last but not least, vildagliptin demonstrated an antihypertensive effect through modulating serum vascular endothelial growth factor (VEGF) in diabetic hypertensive patients. Elevating VEGF levels improve physiological angiogenesis and vasculature [34]. VEGF signaling pathway inhibitors (VSP) have been approved for treatment of a number of different malignancies. One quarter of patients starting VSP therapy struggle with hypertension, and nearly every patient receiving VSP has an increase in blood pressure [122]. Low levels of VEGF may predict poor clinical outcome of CHD patients after PCI treatment [123]. As we mentioned in a previous paragraph, VEGF may increase the risk of plaque rupture, so the influence of elevated VEGF level requires detailed research, especially for patients with both atherosclerosis and hypertension.

## 6. Cardiovascular Outcome

Patients with type 2 diabetes are two-fold to six-fold more prone to cardiovascular disease (CVD) than nondiabetics, making it a leading cause of death in the population. [124] Therefore, the main objective of glycemic control should be preventing death and limiting morbidity due to CVD and microvascular diseases. The earlier registered antidiabetic agents such as biguanides, sulfonylureas (SU), and thiazolidinediones have not been tried for CV safety in large outcome trials. Metformin presented a reduction in CV events in an inadequately powered subgroup with a small number of patients in the The UK Prospective Diabetes Study trial [125]. On the other hand, groups such SGLT-2 inhibitors and GLP-1 agonists revealed themselves as cardioprotective. The SLGT2 inhibitors reduced the risk for a major cardiac event by 11% (hazard ratio, 0.89; 95% CI, 0.83–0.96) in patients with CVD. [126,127]. Metanalysis of seven randomized controlled trials of GLP-1 agonists versus placebo shows that the treatment was associated with a significant 12% lower risk for MACE (cardiovascular death, stroke, or myocardial infarction), a 12% reduction in all-cause mortality risk, and a significant 9% reduction in rates of hospitalization for heart failure [128,129]. 

According to directives of regulatory agencies, it has been required since 2008 that all new antihyperglycemic agents, including DPP-4 inhibitors and GLP-1 agonist, undergo long-term CV safety assessments [130]. Although vildagliptin still lacks established or ongoing randomized controlled trials for CV outcomes, unlike the other DPP-4 inhibitors [131,132,133,134,135,136], a meta-analysis reporting vildagliptin CV safety based on a large pool of double-blind, randomized-controlled phase III and IV vildagliptin studies shows that vildagliptin is not associated with an increased risk of MACE relative to comparators. [137] However, there are reports of anti-sclerotic and vascular protective effects in experimental conditions, clinical efficacy of vildagliptin in prevention of major adverse CV events remains debatable. Patil et al. (2012) comprising 18 randomized trials (*n* = 8544) where patients were randomized to DPP-4 inhibitors (*n* = 4998), including vildagliptin, and other antidiabetic agents such as metformin, SU, and/or placebo (*n* = 3546) showed that only 1 of the DPP4 inhibitors, sitagliptin, presented a significant risk decrease (RR 0.37, *p* = 0.001). Risk decreases achieved with saxagliptin and vildagliptin, although similar in magnitude to that noted with sitagliptin, were statistically insignificant. [138] In another meta-analysis (2013), 70 randomized controlled trials (*n* = 41,959) with DPP-4 inhibitors versus other comparators (oral hypoglycemic agents, insulin, or both) were analyzed and revealed a statistically significant lower risk of MACE, which was consistent across different drugs, although statistical significance was reached only for saxagliptin and vildagliptin [139]. Additional studies are crucial to verify if there is *primus inter pares* among incretin-based therapies or if this is only a class effect.

## 7. Conclusions

**Figure 1:**
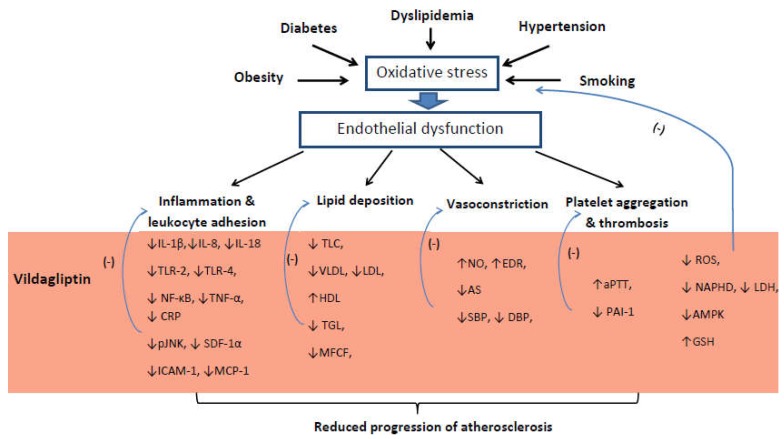
Proposed influence of vildagliptin on atherosclerosis pathophysiology. Note: ↓ = reduction, ↑ = increase, (-) = inhibition, IL-1β = Interleukin 1 beta, IL-8 = Interleukin 8, IL-18 = Interleukin 18, TLR2 = Toll-like receptor 2, TLR4 = Toll-like receptor 4, NF-κB = nuclear factor kappa-light-chain-enhancer of activated B cells, TNF-α = tumor necrosis factor α, CRP = C-reactive protein, pJNK = phosphorylated c-Jun N-terminal kinase, SDF-1α = stromal cell-derived factor 1, ICAM-1 = intercellular adhesion molecule 1, MCP-1 = Monocyte chemoattractant protein-1, TLC = total cholesterol level, VLDL = very low density lipoprotein, LDL = low density lipoprotein, HDL = high density lipoprotein, TGC = triglycerides, MFCF = macrophages foam cells formation, NO = nitric oxide, EDR = endothelial-dependent relaxation, AS – arterial stiffness, SBP = systolic blood pressure, DBP = diastolic blood pressure, aPTT = activated partial thromboplastin time, PAI-1 = plasminogen activator inhibitor-1, ROS = reactive oxygen species, NADPH = Nicotinamide adenine dinucleotide phosphate, LDH = lactate dehydrogenase, AMPK = 5’AMP-activated protein kinase, GSH = glutathione.

The presented results suggest that the benefits of vildagliptin extend far beyond glycemic control. Significant improvement of the impaired endothelial function, reduction of inflammation, and balancing of lipid metabolism disorders allow to regulate the process of atherosclerosis in various stages of its development. Alleviating arterial stiffness, improving endothelium-dependent relaxation, and lowering blood pressure can have an impact in the management of hypertension. If vildagliptin is demonstrated to have clinically meaningful activity in humans, one potential application may be to reduce the burden of certain cardiovascular disorders (Figure 1). However, the presented studies have many limitations. Some of the obtained results like effect of vildagliptin on lipid metabolism are inconclusive. The contradictory results may be due to significant different methodologies such as differences in dosage and time of treatment and a summary of experimental studies, animal models, and human studies together. Promising, vasculoprotective properties of vildagliptin require multidimensional verification in randomized, multicenter studies on large groups of participants.

## Figures and Tables

**Table 1:** Summary of reviewed results.

Authors	Subject of Study	Dose of Vildagliptin	Results
Lee et al. (2016) [13]	LPS stimulated RAW264.7 cells	varied at every stage of the experiment	↓iNOS, ↓ NF-κB, ↓pJNK, ↓TLR-2, ↓TLR-4,
Dei et al. (2017) [14]	rBMVECs	2.5–5 mg/day for 4–12 months	↓ SDF-1α ↓ EPC
Zhang et al. (2018) [15]	HAECs	5 and 10 μM for 24–72 h.	↓ LDH, ↓ ROS, ↓TNF-α, ↓IL-8, ↓ICAM-1, ↓MCP-1, ↓TLR-4, ↓NF-κB
Qi et al. (2019) [16]	HUVECs	2.5 and 5 μM for 24h	↓ LDH, ↓ NAPHD, ↓AMPK, ↓IL-1β, ↓IL-18, ↑eNOS, ↑GSH
Liu et al. (2019) [17]	HUVECs and diabetic mice	varied at every stage of the experiment	↓mtROS, ↑ATP, ↓Drp1
Seo al. (2019) [18]	rabbit aortic rings	varied at every stage of the experiment	↑VD
Oeseburg et al. (2010) [19]	HUVECs and diabetic fatty rats	varied at every stage of the experiment	↑cAMP, ↑PKA, ↑CREB, ↑HO-1
Terasaki et al. (2012 and 2013) [20,21]	Diabetic Apoe (–/–) mice	varied at every stage of the experiment	↓MFCF
Khan et al. (2015) [22]	STZ-induced diabetic rats	10 or 20 mg/kg/day for 3 weeks	↓ TLC, ↓ TGL,↓ CRP ↓TNF-α, ↑aPTT, ↑NO
Jain et al. (2015) [23]	diabetic rats	varied at every stage of the experiment	↑NO, ↑EDR, ↓ROS, ↓MPO, ↑GSH
Koyama et al. (2016) [24]	rabbits	10 mg/kg/day for 5 weeks	↑eNOS, ↑VGIH
Zhang et al. (2018) [25]	diabetic rats	10 or 20 mg/kg/day for 12 weeks	↓ TCL, ↓ ED, ↓Angptl3, ↓Bhmt,↓ Pon1
Ji et al. (2019) [26]	diabetic mice	35 mg/kg/day for 4 weeks	↓ ERS, ↓ NF-κB,
van Poppel et al. (2011) [27]	DM2 patients	50 mg /day for 4 weeks	↑EDR
Noguchi et al. (2015) [28]	normoglycemic patients	50 mg once	↓ TGL, ↓EDs
Tani et al. (2015) [29]	DM2 patients	50 mg/day for 8 weeks	↓ PAI-1
Evans et al. (2016) [30]	DM2 patients	50 mg once or twice/day for 24 weeks	↓SBP, ↓ DBP, ↓TGL, ↓VLDL,↓LDL, ↑HDL
Duvnjak et al. (2016) [31]	DM2 patients	100 mg/day for 12 weeks	↓TLC,↓LDL, ↓hsCRP, ↓AS, ↓CBP
Park et al. (2017) [32]	DM2 patients	1 mg/twice a day for 12 weeks	↓SDF-1α
Younis et al. (2017) [33]	patients with DM2 and CAD	Metformin + vildagliptin 25 or 50 mg/day	↓ IL-1β, ↓hsCRP
El-Naggar et al. (2019) [34]	DM2 patients with hypertension	50 mg/twice a day + 25 mg/day captopril for 24 weeks	↓BP,↓VEGF

Note: ↓ = reduction, ↑ = increase, p- = phosphorylation, LPS = Lipopolysaccharides, iNOS = inducible nitric oxide synthase, NF-κB = nuclear factor kappa-light-chain-enhancer of activated B cells, JNK = c-Jun N-terminal kinase, TLR2 = Toll-like receptor 2, TLR4 = Toll-like receptor 4, rBMVECs = rat brain microvascular endothelial cells, SDF-1α = stromal cell-derived factor 1α, EPC – endothelial progenitor cells, HAECs = Human aortic endothelial cells, LDH = lactate dehydrogenase, ROS = reactive oxygen species, TNF-α = tumor necrosis factor α, IL-8 = Interleukin 8, ICAM-1 = intercellular adhesion molecule 1, MCP-1 = Monocyte chemoattractant protein-1, HUVECs = Human umbilical vein endothelial cells, NADPH = Nicotinamide adenine dinucleotide phosphate, AMPK = 5’AMP-activated protein kinase, IL-1β = Interleukin 1 beta, IL-18 = Interleukin 18, eNOS = endothelial nitric oxide synthase, GSH = glutathione, mtROS –mitochondrial reactive oxygen species, ATP = adenosine triphosphate, Drp1 = Dynamin related protein 1, VD = vascular dilatation, cAMP = 3’,5’-cyclic adenosine monophosphate, PKA = protein kinase A, CREB = cAMP response element-binding protein, HO-1 = Heme oxygenase 1, MFCF = macrophages foam cells formation, STZ = streptozocin, TLC = total cholesterol level, TGC = triglycerides, CRP = C-reactive protein, aPTT = activated partial thromboplastin time, NO = nitric oxide, EDR = endothelial-dependent relaxation, MPO = myeloperoxidase, VGIH – vein intimal grafts hyperplasia, ED = endothelium dilatation, Angptl3 = Angiopoietin-like 3, Bhmt = betaine-homocysteine S-methyltransferase, Pon1 = paraoxonase 1, ERS = endoplasmic reticulum stress, DM2- diabetes mellitus type 2, EDs = endothelial dysfunction, PAI-1 = plasminogen activator inhibitor-1, SBP = systolic blood pressure, DBP = diastolic blood pressure, VLDL = very low density lipoprotein, LDL = low density lipoprotein, HDL = high density lipoprotein, hs CRP = high specific C-reactive protein, AS = arterial stiffness, CBP = central blood pressure, SDF-1α = stromal cell-derived factor 1, CAD = coronary artery disease, VEGF = vascular endothelial growth factor.
